# Assemblages of Ground Beetles (Coleoptera, Carabidae) in Secondary Deciduous Forests of Selected Regions of the Nemoral Biome in European Russia (Spring—Early Summer Aspect)

**DOI:** 10.3390/insects17070724

**Published:** 2026-07-13

**Authors:** Victor V. Aleksanov, Sergey K. Alekseev, Alexander B. Ruchin, Mikhail N. Esin, Sergei V. Lukiyanov, Evgeniy A. Lobachev

**Affiliations:** 1 Parks Directorate of Kaluga Region, 248023 Kaluga, Russia; victor_alex@list.ru (V.V.A.); stenus@yandex.ru (S.K.A.); 2 Joint Directorate of the Mordovia State Nature Reserve and National Park “Smolny”, 430005 Saransk, Russia; esinmishka@gmail.com (M.N.E.); lukiyanovs@gmail.com (S.V.L.); lobachevea@ya.ru (E.A.L.)

**Keywords:** ground beetles, Carabidae, secondary forest, nemoral forests, oak forests, ash forests, forest-steppe, activity density, similarity, life forms

## Abstract

The nemoral biome encompasses some of the most diverse and productive ecosystems in Europe, yet it has been extensively transformed by human activity. The capacity of secondary forests that have developed on sites of clear-cut broadleaved forests to sustain ground beetle diversity remains insufficiently studied. Ground beetles were sampled using pitfall traps during May–July in 21 post-logging forest stands across six administrative regions of Central Russia. A total of 90 ground beetle species were recorded. The superdominant species in most habitats were *Carabus cancellatus* Ill. and *Pterostichus melanarius* Ill. The median species richness per sampling plot was 17, and the median activity density was 33.6 individuals per 100 trap-days. The similarity of ground beetle assemblages, in terms of both species composition and community structure, is determined primarily by habitat characteristics rather than by the spatial proximity of sampling sites. The most robust predictor of both species composition and community structure was the percentage cover of *Corylus avellana*. A large proportion of exposed soil within forest stands is associated with a higher activity density of mixophytophagous stratohortobionts, predominantly *Harpalus rufipes* L. As the distance from large forest tracts increased, the activity density of large walking epigeobiont zoophages declined. Overall, the species structure of ground beetle assemblages was only weakly predictable.

## Simple Summary

We investigated ground beetles in deciduous forests that developed on clear-cut sites within the broadleaved forest and forest-steppe subzones across six administrative regions of Central Russia using pitfall traps during May–July. The similarity of ground beetle assemblages is more strongly determined by specific habitat characteristics, particularly by the percentage of hazel in the shrub layer, rather than by the spatial proximity of habitats. A high proportion of exposed soil within forests may lead to an increase in the activity density of certain species typical of open habitats. As the distance from large forest tracts increases, the activity density of the large walking epigeobionts declines.

## 1. Introduction

Anthropogenic activity over many centuries has led to the disruption of natural processes of forest ecosystem recovery [1]. However, the main trend of global changes in forest ecosystems emerged and has been occurring over the last 150–200 years, when timber resources began to be exploited much more intensively [2,3,4,5,6]. These disturbances are manifested in changes in the development of the tree layer and in tree generation turnover, in the spatial mosaic structure of ecosystems and their temporal dynamics, and in the functioning of all forest layers and components [7,8,9,10]. For virtually any type of intensive human use of natural forest ecosystems, it can be stated that it leads to the simplification of biota and the loss of existing biodiversity [11,12,13]. At the same time, native forest ecosystems lose part of their properties and functions, as well as their natural structural and dynamic features [14,15,16]. Forest ecosystems that have been transformed in the past by intensive human economic activity and have not yet regained their natural structural and dynamic organization are referred to as secondary forests [15,17]. Such secondary forests, for example, arise on clear-cut areas or abandoned agricultural clearings [14,18,19]. The distinction between natural and secondary forests is rather conditional, since many “natural” forests may have been destroyed several centuries ago and only later recovered [1].

While natural forests are characterized by a complex mosaic structure of the tree canopy (gaps, edges, standing dead trees, coarse woody debris, etc.), secondary forests are more often characterized by the presence of clearly defined closed stands, the formation and development of which are driven by past large-scale disturbances. Such forests usually contain few shrubs, and the herbaceous layer is sparse [20,21,22,23]. In low-disturbed natural forests, trees of all ages and size classes are typically present, whereas secondary forests are characterized by the dominance of a single cohort and a narrow range of tree size classes. In deciduous forests, such stands often develop on former clear-cut areas from remaining stumps. This is called sprout regeneration of forest ecosystems [24,25].

Ground beetles (Coleoptera: Carabidae) are reliable indicators of environmental conditions and are sensitive to changes in the environment [26,27]. The differentiation of ground beetle assemblages reflects the classification of land units, and differences in species structure are correlated with variation in several plot characteristics, including canopy and ground cover, vegetation structure, tree density, leaf litter depth, and soil moisture [28].

In particular, coniferous stands in boreal forests contain fewer species and individuals and differ in species composition from mixed wood forests and deciduous stands, where leaf litter is present [29]. Accordingly, ground beetle assemblages respond rapidly to increased forest management intensity and may reflect the history of forestry practices [30] and can be used as indicators of sustainable forest management [31].

The most important factor in the transformation of ground beetle populations in forests is clear-cut logging followed by the development of secondary forests. It is known that shortly after logging, both species richness and abundance of ground beetles increase, mainly due to the appearance of species typical of open habitats [32,33,34,35,36,37]. As the clear-cut area becomes overgrown and canopy closure increases, species richness declines [38,39,40]. At the same time, the trajectories of changes in individual characteristics of carabid assemblages may be non-monotonic and differ among forest types. For example, in boreal forests, the highest ground beetle abundance was observed immediately after logging and in mature forests [41], whereas in forest-steppe oak forests it occurred in clear-cuts and in mid-successional stands [42].

Although logging leads to a short-term increase in species richness at a local scale, at the landscape level, a negative effect on rare ground beetle species has been recorded, as this form of forest use leads to a reduction in open habitats within forests [43]. This corresponds to the generally lower structural diversity of secondary post-logging forests [44]. At the same time, it is known that local differences, determined, for example, by soil properties, have a stronger influence than the effects of logging. For instance, in Białowieża, it was found that managed forests do not differ from primeval forests in species richness, although they have lower Shannon diversity, and that differences between ground beetle assemblages are more strongly determined by soil properties [45].

The cited and other studies addressing the effects of clear-cut logging on ground beetles have been concentrated predominantly in the boreal and hemiboreal biomes, since these regions contain the main areas of commercially exploited temperate forests. Considerably less attention has been paid to understanding ground beetle assemblages in secondary forests of other biomes.

The nemoral biome encompasses territories with a temperate climate and sufficient moisture and is characterized by the dominance of deciduous broadleaved trees; it represents the warmest forest regions of the temperate zone [46]. It contains some of the most productive ecosystems in Europe. The nemoral biome includes the broadleaved forest and forest-steppe subzones [47]. In Europe, it partly corresponds to the Continental Biogeographical Region [48]. The nemoral biome has been strongly transformed by human activity; therefore, its forest area is small in comparison with the boreal and hemiboreal biomes [44]. The nemoral biome also includes central parts of the ranges of several ground beetle species, including vulnerable species [49].

Information on ground beetles in the nemoral biome of the East European Plain has been presented in various studies, both for the forest-steppe [50,51,52,53,54,55,56,57,58] and for broadleaved forests [59,60,61,62]. There has been experience in comparing forests of these subzones (treated as different natural zones), which revealed similarity in dominant species both between these subzones and with more northern regions [42]. A substantial body of data has been published for administrative regions encompassing both subzones of the nemoral biome [63,64]. Information is also available on carabid assemblages in oak forests of different ages in Voronezh Oblast [53]. Broadleaved forests have been compared along an urbanization gradient [65]. Attempts to identify general patterns of secondary forests have been undertaken for the territory of Mordovia [66]. However, we are unaware of any attempts to analyze ground beetle assemblages in secondary forests across different regions.

Post-logging nemoral forests, in contrast to old-growth forests, are characterized by a low stock of organic matter, even-aged stand structure, and simple spatial organization [44,67]. This indicates a limited number of resources and restricted opportunities for the differentiation of ecological niches of ground beetles [68,69]. Therefore, reduced species richness and abundance of these beetles may be expected. Dense and homogeneous stands lacking gaps in the tree canopy are likely to be unfavourable for inhabitants of open habitats and for mixophytophagous species (in terms of the classification of life forms proposed by Sharova [70]) associated with open microhabitats within forests [71]. At the same time, mixophytophages are characteristic of clear-cut patches [42]; therefore, the persistence of some of these species in secondary forests cannot be excluded.

The aim of our study was to investigate ground beetle assemblages in forests of secondary origin across different regions of the nemoral biome in the central part of the East European Plain. The following objectives were addressed: (1) to determine the species composition of ground beetles in secondary forests and identify dominant species, and to assess the species diversity, activity density, and life-form composition of ground beetles in secondary forests of the nemoral biome in spring and summer season; (2) to assess the degree of similarity among different ground beetle assemblages in terms of species composition and structure, to test whether this similarity corresponds to spatial proximity, and to evaluate which habitat parameters determine the differentiation of ground beetle assemblages in secondary forests.

Accordingly, we tested the following hypotheses: (1) secondary forests should be dominated by relatively large forest generalists (eurytopic forest species) belonging to the zoophages in terms of Sharova’s classification of life forms (such species are expected to colonize clear-cut areas more rapidly, whereas mixophytophagous species are disadvantaged by dense and homogeneous stands suppressing the development of herbaceous vegetation). (2) Similarity of ground beetle assemblages among different regions in terms of species composition should reflect their spatial proximity (because each region possesses its own regional species pool), whereas similarity in species structure (i.e., dominant species composition) should reflect habitat characteristics associated with the tree layer, shrub layer, and ground vegetation.

## 2. Materials and Methods

### 2.1. Study Area and Sample Plots

**Figure 1:**
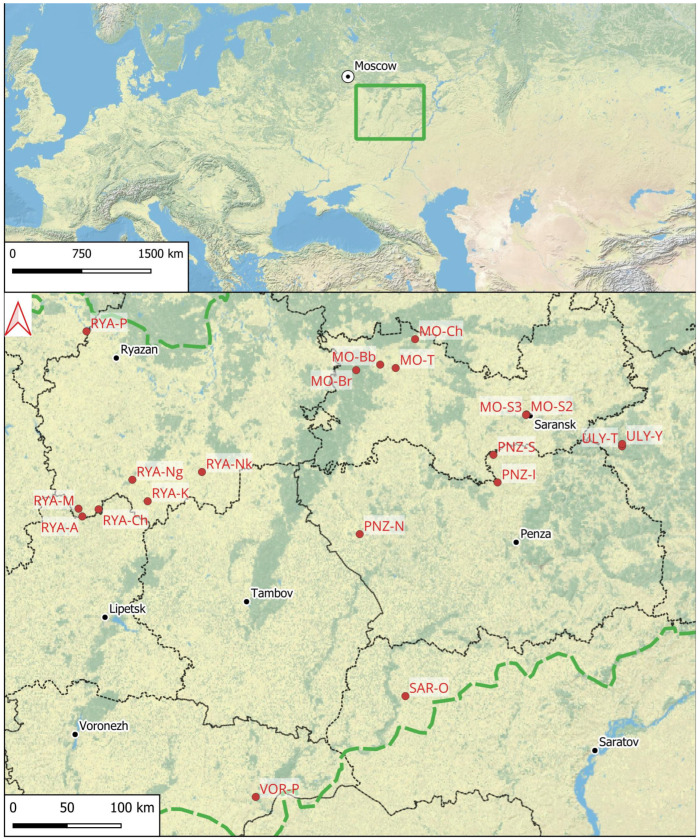
Map of the sampling plots: thick green dashed line—boundaries of the nemoral biome; thin grey dashed line—boundaries of administrative regions.

The study area is located between 51.151° and 54.831° N latitude and between 39.245° and 46.393° E longitude (Figure 1). Administratively, it includes the Republic of Mordovia and the Penza, Ryazan, Saratov, Ulyanovsk, and Voronezh regions (European Russia). The territory belongs to the basins of the Don, Oka, and Sura rivers. The relief is predominantly flat. The climate is moderate continental.

In native forests, the first canopy layer is formed by pedunculate oak *Quercus robur* L., ash *Fraxinus excelsior* L., and small-leaved lime *Tilia cordata* Mill. Smaller trees include elm *Ulmus glabra* Huds., Norway maple *Acer platanoides* L., and field maple *Acer campestre* L. The shrub layer is dominated by common hazel *Corylus avellana* L.; typical species also include *Euonymus verrucosus* Scop., *Lonicera xylosteum* L., *Prunus padus* L., and *Frangula alnus* Mill. Pine forests dominated by Scots pine, *Pinus sylvestris* L., are widespread on sandy deposits.

In secondary forests, along with tree species characteristic of native forests, silver birch *Betula pendula* Roth and aspen *Populus tremula* L. are present. *Acer platanoides* frequently reaches the first canopy layer, sometimes together with *Ulmus glabra*, and pine may also occur as an admixture. In the shrub layer of some forests, *Sorbus aucuparia* L. and *Salix caprea* L. are also present; occasionally, escaped cultivated species such as *Malus domestica* (Suckow) Borkh. or *Sorbaronia* × *fallax* (C.K.Schneid.) C.K.Schneid. occur.

**Figure 2:**
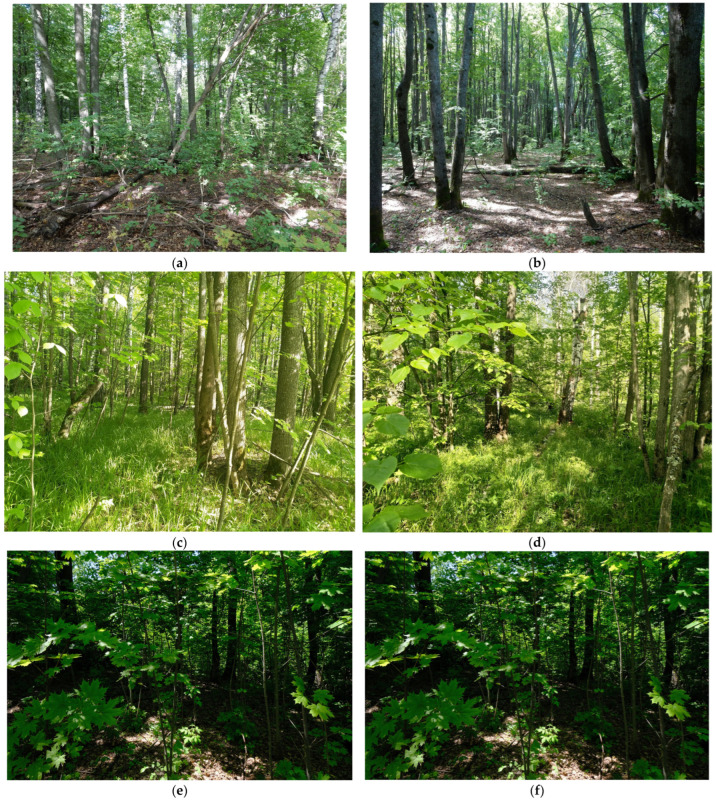
Photographs of selected sampling plots. (**a**) RYA-M; (**b**) RYA-P; (**c**) ULY-T; (**d**) ULY-Y; (**e**) SAR-O; (**f**) VOR-P.

A total of 21 secondary forest sites were surveyed (Table 1, Figure 2, Table S1). In our study, secondary deciduous forests were predominantly coppice forests of secondary origin (island-type stands) that developed on sites of destroyed or heavily altered broadleaved forests following logging. The composition of the tree canopy and regeneration layer is presented in Table 1. The abundance of each tree canopy and understory species was estimated as percentage cover (Table S2).

Ground cover was characterized by the percentage of bare ground, the area occupied by projections of aboveground parts of grasses and sedges, and the projections of forbs. Among sedges, *Carex pilosa* Scop. was the most common species, whereas among grasses, *Milium effusum* L. predominated. Typical nemoral forbs recorded in the sampling plots included *Aegopodium podagraria* L., *Convallaria majalis* L., *Stellaria holostea* L., *Pulmonaria obscura* Dumort., and *Galium odoratum* (L.) Scop.

### 2.2. Data Sampling

Material was collected using soil pitfall traps during May–July in 2008, 2014, 2015, 2019, 2022, and 2023. The traps consisted of 0.5 L plastic cups with a trap opening diameter of 87 mm; a 4% formalin solution was used as a preservative. At each sampling plot, 10 traps were installed in a single line with a spacing of 2–3 m between adjacent traps.

In total, 6510 adult specimens representing 90 species were recorded. Species abundance was assessed as the activity density, expressed as the number of specimens per 100 trap-days.

Species identification of ground beetles was performed using identification keys [72,73,74]. Nomenclature follows the Catalogue of Palaearctic Coleoptera [75]. Classification of life forms was based on Sharova [70]. Total abundance was calculated for life-form classes—zoophagous and myxophytophagous groups—as well as for selected life-form categories, including large walking epigeobiont zoophages, myxophytophagous geohortobionts, and myxophytophagous stratohortobionts. Range types were analyzed only for *Carabus* species, for which they are clearly defined [49]. Species with West and Central European ranges were classified as “western”, species with East European and East European–Siberian ranges as “eastern”, and species with European, Sibero-European, and Asiatic-European ranges as widely distributed.

Habitat preference was characterized according to published data [42,76,77]; meadow, meadow-field, and field species were treated as inhabitants of open habitats. To identify specific features of the studied forests, comparisons were made with published data from low-disturbed forests of the biome collected using the same methods.

### 2.3. Data Analysis

Data analysis was performed in R version 4.5.2 [78].

Species accumulation curve with the calculation of estimated species number was performed in the *vegan* package (version 2.7-5) [79]. Sample coverage was calculated in the iNEXT package (version 3.0.2) [80].

The *vegan* package was used for calculating and analyzing similarity matrices [79]. Similarity in species composition of assemblages (faunistic similarity) was assessed using the Jaccard index in its binary form, whereas similarity in species structure was assessed using the abundance-based Jaccard index. Ordination was performed using non-metric multidimensional scaling (NMDS; function *isoMDS*).

To evaluate the influence of environmental factors on the differentiation of ground beetle assemblages (similarity in species composition and species structure), permutational multivariate analysis of variance (PERMANOVA; function *adonis2*) was applied. The tested predictors included key habitat characteristics reflecting the representation of major tree species in the canopy and regeneration (undergrowth) layer, as well as ground cover conditions: oak in the canopy (oak_t), oak in the regeneration layer (oak-un), ash in the canopy (ash_tree), ash in the regeneration layer (ash_un), hazel in the shrub layer (*Corylus*), proportion of bare ground, proportion of forbs, and the Year. The proportion of grasses and sedges in the ground layer was excluded from the analysis due to its correlation with hazel abundance (Pearson’s *r* = 0.594, *p* = 0.0057). Non-significant variables were subsequently removed from the model. Climate variables (mean temperature and precipitation for May–June) were extracted from NASA POWER for each site-year combination based on geographic coordinates [81]. These variables were used to assess whether interannual variation in community composition could be attributed to climatic differences.

To evaluate the influence of individual sampling sites on the PERMANOVA results, we conducted a leave-one-site-out sensitivity analysis. Each sampling site was sequentially excluded, the corresponding subset of the Jaccard dissimilarity matrix and environmental data was analyzed, and the PERMANOVA model was refitted using the same predictors and 999 permutations. The stability of predictor significance (*p*-values) and effect sizes (partial R^2^) was assessed across all iterations.

Relationships between individual characteristics of ground beetle assemblages and environmental parameters were assessed using Spearman’s rank correlation coefficient, as the relationships were non-linear. Diversity indices were calculated in PAST 5 [82]. Shannon diversity indices were compared using a *t*-test. Mapping and spatial analyses were performed in QGIS 3. Distances and areas were calculated using the WGS 84 ellipsoid (EPSG: 7030). The position of each sampling plot within the landscape was characterized by its distance to large forest tracts (defined as forest areas exceeding 1 km^2^).

## 3. Results

### 3.1. General Characteristics of Species Composition

**Figure 3:**
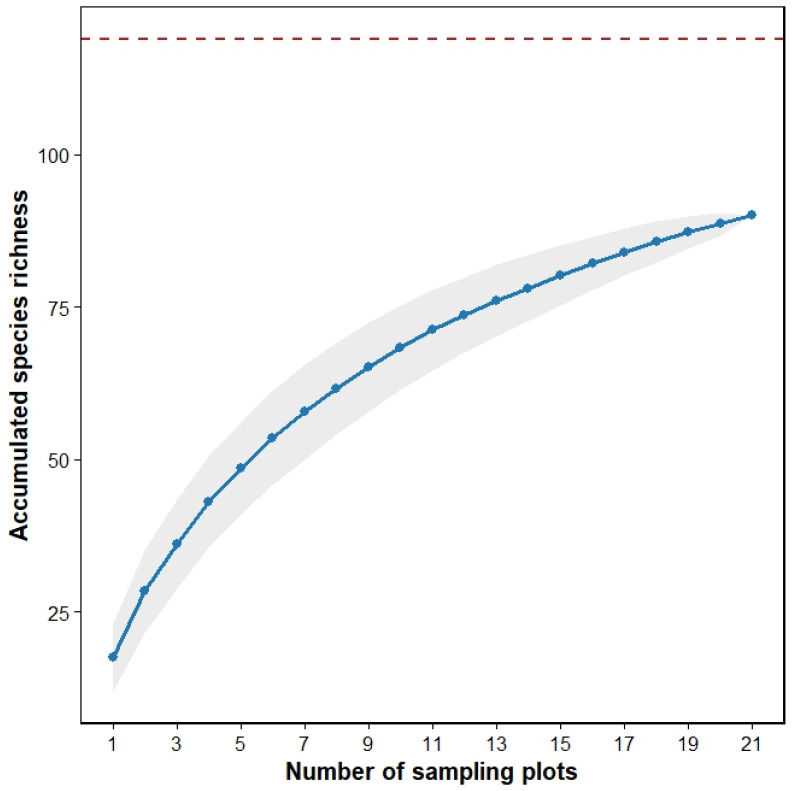
Species accumulation curve for carabids in secondary deciduous forests across some regions of Russia within the nemoral biome. The red line is expected species richness according to The Chao estimator.

**Figure 4:**
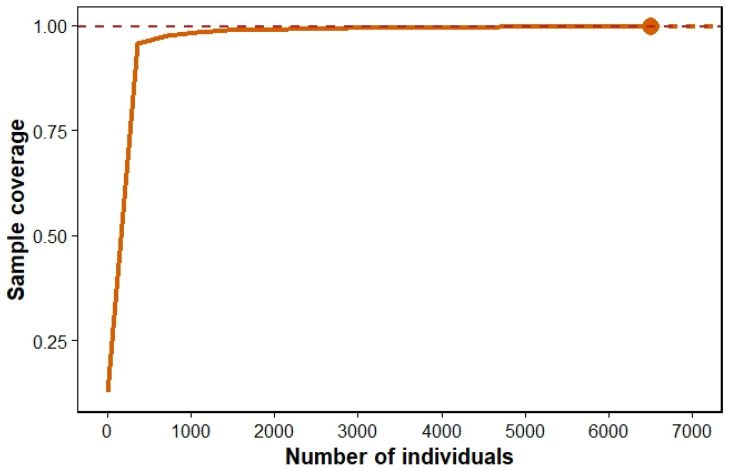
Sample coverage for carabids in secondary deciduous forests across some regions of Russia within the nemoral biome.

A total of 90 ground beetle species belonging to seven subfamilies were recorded. None of the species was present on all sampling plots (Table 2). The Chao estimator predicted approximately 119 species, so the completeness consists 76%, suggesting that many species of secondary deciduous forests may remain undetected (Figure 3). However, the sample coverage was very high (SC = 0.997), indicating that nearly all individuals in the assemblage belonged to species already represented in the sample (Figure 4). This discrepancy reflects the relatively high number of singleton species (22 species represented by a single individual and 14 species represented by 2–4 individuals), whereas the probability of encountering additional abundant species is negligible. Thus, although additional sampling would likely reveal a limited number of additional rare species, the current sampling adequately characterized the dominant component of the carabid assemblages of secondary deciduous forests among the nemoral biome.

The most frequent and dominant species across the vast majority of plots were *Carabus cancellatus* Illiger, 1798, and *Pterostichus melanarius* (Illiger, 1798). These species showed a moderate positive correlation in their distribution among plots (Spearman’s *r* = 0.49, *p* = 0.024).

The superdominant species occurring in five or more sampling plots included *Carabus granulatus* Linnaeus, 1758, *Pterostichus oblongopunctatus* (Fabricius, 1787), *Limodromus assimilis* (Paykull, 1790), and *Harpalus rufipes* (De Geer, 1774). In individual forests, dominant species included *Calosoma inquisitor* (Linnaeus, 1758), *Poecilus versicolor* (Sturm, 1824), *Pterostichus strenuus* (Panzer, 1796), *Harpalus tardus* (Panzer, 1796), and *Amara communis* (Panzer, 1797). *Pterostichus niger* (Schaller, 1783) had a high occurrence frequency but was not among the superdominants, becoming dominant in only three habitats. Traditionally, most of the listed species are considered forest dwellers, whereas species of open habitats include *P. versicolor*, *H. rufipes*, *H. tardus*, and *A. communis*. *Carabus cancellatus* is a forest species in the forest-steppe zone but tends to be associated more with open habitats in northern regions. *Pterostichus melanarius* is sometimes regarded as an eurytopic species [42,76,77]. Notably, one of the commonest species of the broadleaved forest [42,49,77], *Carabus granulatus*, was relatively abundant only in three habitats. Overall, in addition to forest species (including forest–wetland species) and open-habitat species, a small number of riparian species were also recorded, such as *Bembidion guttula* (Fabricius, 1792) and *Chlaenius nigricornis* (Fabricius, 1787).

In terms of life forms, dominant species are characterized as large walking epigeobiont zoophages and soil–litter stratobionts, with participation of litter-dwelling stratobionts, dendroepigeobionts, myxophytophagous geohortobionts, and stratohortobionts.

Analyzing the chorotypes of *Carabus* species, we found that the fauna is relatively uniform across the study area. There was no significant correlation between longitude and the presence of western species (Pearson’s *r* = −0.149, *p* = 0.5299), nor between longitude and the number of eastern species (Pearson’s *r* = 0.174, *p* = 0.4619). The only western species, *Carabus nemoralis* O.F. Müller, 1764, was absent from only the two easternmost plots. Among the eastern species characteristic of the East European forest-steppe [49,51,83], the forest species *Carabus marginalis* Fabricius, 1794 was relatively abundant in only a single forest in the southern part of the study area (Voronezh Region), whereas the meadow species *Carabus violaceus aurolimbatus* Dejean, 1830 was recorded in small numbers in only two habitats in the western part of the study area (Ryazan Region). *Carabus schoenherri* Fischer von Waldheim, 1820 was found exclusively in the eastern part of the study area (Ulyanovsk Region), whereas *Carabus stscheglowi* Mannerheim, 1827 occurred only in two of the southernmost habitats. In all plots, species with wide distributions (Asiatic–European, Sibero-European, or European ranges) were dominant.

Among individual sample plots, activity density of ground beetles showed a very wide range, from 6.7 to 137.4 individuals per 100 trap-days, with a mean of 52.0 and a median of 33.6. Only four sampling sites exceeded 100 individuals per 100 trap-days (Table 3). Species richness per sampling plot varied from 10 to 32 species (excluding MO-Br as an outlier), with a mean of 17 and a median of 18. The degree of dominance of the most abundant species (Berger–Parker index) is very high, averaging 39%, and exceeding 50% in four sampling sites. Shannon diversity index values are generally low, exceeding two only in nine sampling sites.

### 3.2. Differentiation of Ground Beetle Assemblages by Species Composition and Structure

Similarity among the studied forests in terms of both species composition and community structure of ground beetles was extremely low: the mean value of the binary Jaccard similarity index was 0.25, with a maximum of 0.48; the abundance-based similarity index ranged from 0.14 to 0.52.

**Figure 5:**
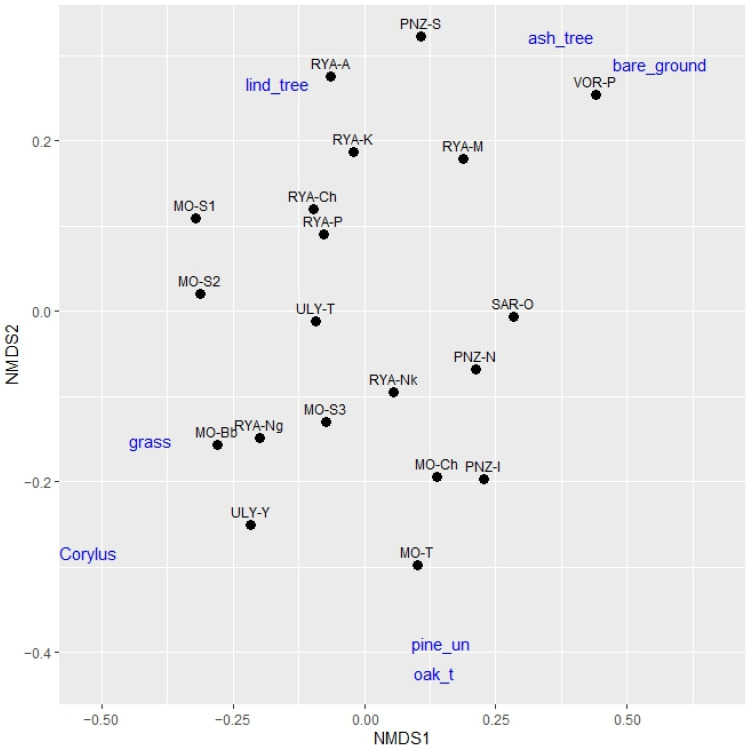
NMDS ordination plot of carabid assemblages. Jaccard index for binary data. Plot labels see Table 1. Factors: bare_ground—percentage of bare ground, grass—percentage of grass and sedges, ash_tree—percentage of *Fraxinus excelsior* in tree layer, lind_tree—percentage of *Tilia cordata* in tree layer, oak_t—percentage of *Quercus robur* in tree layer, pine_un—pine in undergrowth. Corylus—percentage of *Corylus avellana*.

**Figure 6:**
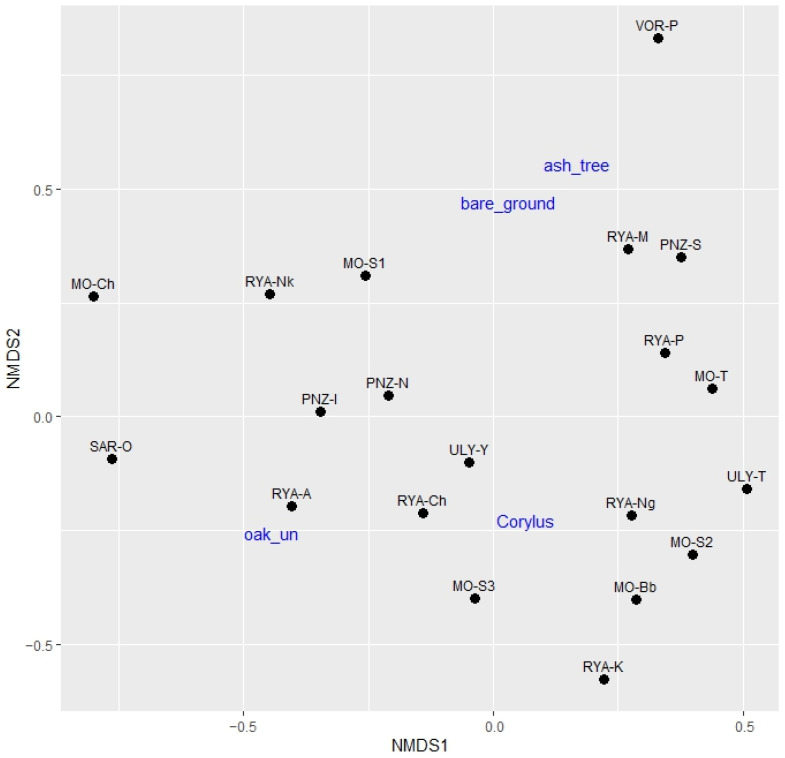
NMDS ordination plot of carabid assemblages. Jaccard index for non-binary data. Designations see Figure 2.

Ordination in NMDS space revealed a pronounced deviation of plot MO-Br from all other biotopes, characterized by very low activity density and species richness of ground beetles. Therefore, this plot was excluded from further analysis. The ordination results are presented in Figure 5 and Figure 6.

Similarity of ground beetle assemblages, both in species composition (binary Jaccard index) and in species structure (abundance-based Jaccard index), does not reflect the spatial proximity of sampling plots. Only isolated pairs of neighbouring sites showed similar assemblages (some sites in the Ryazan Region in terms of species composition and sites in the Penza Region in terms of species structure).

Overall, ground beetle assemblages in terms of species composition form a cloud-like pattern, with one end associated with sites characterized by ash in the canopy layer and exposed soil, and the opposite end associated with sites with high abundance of hazel in the shrub layer and grasses and sedges in the herb layer. The second NMDS axis separates, on the one hand, lime- and ash-dominated forests, and on the other hand, oak forests and sites with pine in the regeneration layer, and can be interpreted as a gradient of light availability in the lower vegetation layers.

In the ordination diagram based on species structure (Figure 5), environmental gradients are less distinct, although an ash-dominated forest in the Voronezh Region (VOR-P), lacking hazel and with a high proportion of bare ground, is clearly separated.

Permutational Multivariate Analysis of Variance showed that the studied vegetation characteristics make a relatively small contribution to the differentiation of ground beetle species composition (binary Jaccard index). Among them, the highest contribution was associated with hazel abundance and oak abundance in the canopy layer; however, ash abundance in the canopy layer, oak abundance in the regeneration layer, and ash abundance in the regeneration layer were also significant (Table 4). The year of study had a relatively large and significant contribution.

Leave-one-site-out analyses indicated that the effects of *Corylus* and Year were robust to the exclusion of individual sampling sites: the first stayed significant in all 20 iterations, and the last was a marginally non-significant (*p* = 0.051) in one iteration (exclusion of MO-S1). The effect of oak abundance in the canopy layer was significant in 19 of 20 iterations. On the contrary, the contribution of ash abundance in the canopy layer became very small after exclusion of VOR-P or MO-Bb, as well as the contribution of oak abundance in the regeneration layer.

To check if interannual differences are determined by climatic conditions, we included average temperature and precipitation in May–June for each site and studied the year in the model (Table 4). In sites surveyed in 2022, the weather of May and June was cooler than in other years (Table S1). However, neither average temperature nor precipitation was found as a significant predictor for carabid assemblages (Table 4).

For the differentiation of ground beetle species structure (abundance-based Jaccard index), only hazel abundance was found to be significant (Table 5). Leave-one-site-out analyses indicated that the effect of *Corylus* was robust in all 20 iterations. Exclusion of the Year variable leads to the significant effect of oak abundance in the regeneration layer, but leave-one-site-out analyses show that this effect was significant only in five of 20 iterations.

### 3.3. Distribution of Some Groups of Life Forms and Carabid Species Among the Studied Forests

When describing ground beetle assemblages using formal structural characteristics, it should be noted that these characteristics do not always reflect the same biological meaning. For example, the elevated species richness observed at RYA-M is attributable to the presence of open-habitat species. At four of the seven sites where total ground beetle number exceeded 75 individuals (PNZ-S, RYA-M, RYA-P, and VOR-P), this pattern was largely determined by the superdominance of the field species *Harpalus rufipes*. The high diversity of ULY-Y compared with the neighbouring ULY-T, despite equal species richness in both habitats, was caused by a multiple decrease in the activity density of all dominant large and medium-sized ground beetle species.

Nevertheless, among all studied sampling plots, the “best” ground beetle assemblage in terms of species richness, diversity, and total activity density was recorded in the ash-dominated forest within a large forest tract in the southern part of the biome (VOR-P).

In this regard, relationships between selected characteristics of ground beetle assemblages and environmental parameters were analyzed. Positive and noteworthy correlations were detected in two cases. The activity density of myxophytophagous species was positively correlated with the proportion of bare ground (*r* = 0.682, *p* = 0.0007), but only due to stratohortobionts, for which the relationship was even stronger (*r* = 0.749, *p* = 0.0001), whereas no correlation was found for geohortobionts (*r* = 0.038, *p* = 0.8736).

**Figure 7:**
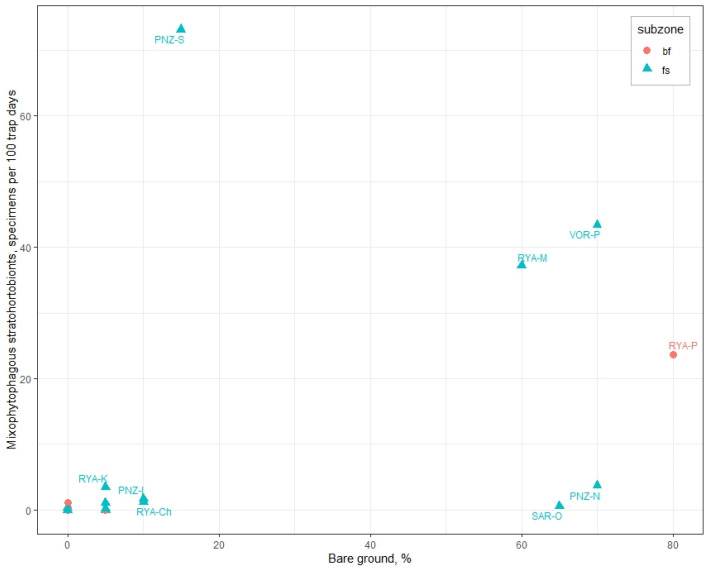
Activity density of myxophytophagous stratohortobionts depends on the percentage of bare ground. Subzones: bf—broadleaved forests, fs—forest-steppe.

Considering the group of myxophytophagous stratohortobionts, it should be noted that this effect was driven almost entirely by the only abundant representative of this group, *Harpalus rufipes* (and to a lesser extent by the closely related *H. griseus*, which became dominant in one habitat). When the proportion of bare ground was below 15%, these ground beetles were almost absent from forests, whereas at higher values their activity density could become high (Figure 7).

**Figure 8:**
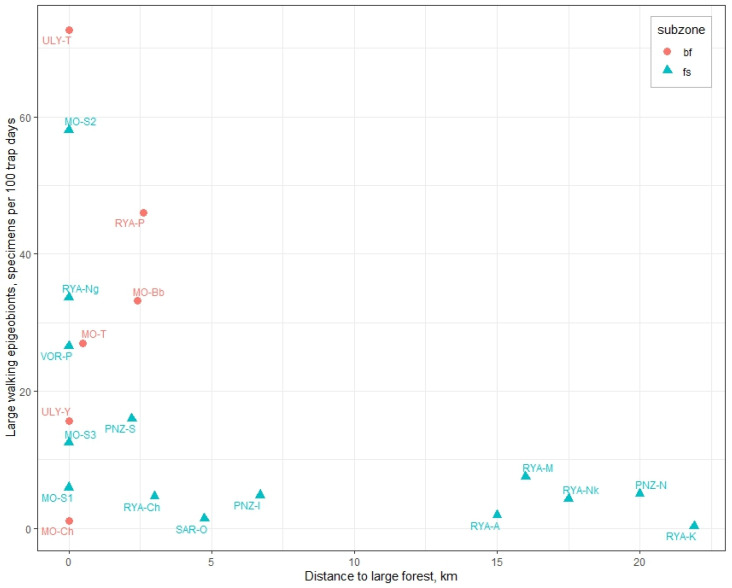
Activity density of large walking epigeobionts depends on the distance to a large forest. Designations see Figure 4.

The activity density of large walking epigeobiont zoophages was negatively correlated with the distance of a sampling plot from the nearest large forest tract (*r* = −0.579, *p* = 0.0075). At sites adjacent to large forest tracts, the activity density of this group varied greatly; however, at distances of 3 km or more, it was consistently very low (Figure 8). The decline in the activity density with increasing distance was especially pronounced within the forest-steppe subzone.

Species richness of epigeobionts itself did not depend on distance. However, remote (isolated) secondary forests were inhabited predominantly by relatively eurytopic species such as *Carabus cancellatus*, *C. convexus*, and *C. nemoralis*. Among individual species, a decrease in the activity density with increasing distance from large forest tracts was detected only for *C. granulatus* (*r* = −0.44, *p* = 0.05). The subendemic forest-steppe forest species *C. marginalis* and *C. stscheglowi* were relatively abundant only in the forest adjoining a large forest tract (VOR-P), although single individuals were also recorded in distant forest patches.

## 4. Discussion

### 4.1. Peculiarities of Species Composition and Species Structure

The results of this study demonstrated a high degree of dissimilarity among ground beetle assemblages in secondary deciduous forests of the nemoral biome. Differences in species composition among secondary forests may partly be explained by regional differences in the species pool [84]. For example, some species occur only in the eastern part of the study region, whereas others are restricted to its southern part. In contrast, some species become progressively rarer toward the south or east of the study area. However, these species do not determine the overall structure of the ground beetle assemblages. More likely, differences in assemblage structure are largely determined by both the history of individual sites and the composition of the surrounding landscape (e.g., forests, agricultural fields, and other habitat types), which influence opportunities for colonization and the success of dispersal from neighbouring habitats [85]. Sample completeness in our survey was relatively low. Nevertheless, compiling a complete list of carabid species occurring in secondary forests across a large region is an unrealistic objective because secondary forests themselves are highly heterogeneous, and the possible combinations of surrounding habitats are virtually unlimited. Consequently, the probability of detecting previously unrecorded rare species remains high. Despite this, the high sample coverage provides a reliable basis for discussing the patterns of dominant species.

In most cases, the core of ground beetle assemblages is formed by *Carabus cancellatus* and *Pterostichus melanarius*. These species inhabit a wide range of habitats and may be characterized as forest generalists or eurytopic species [42,76,77]. The former, being a mesoxerophilous species [49], more strongly reflects the specificity of the eastern sector of the nemoral biome. Both species, in addition to forests, successfully inhabit agrocenoses [86,87,88,89,90]. Their dominance in secondary forests may reflect both adaptation to harsh conditions (absence of dense nemoral herb vegetation, low amounts of litter and dead wood) [89,90] and the use of surrounding agrocenoses.

*Carabus cancellatus* is capable of considerable walking dispersal and may also climb vegetation while hunting [49]. *Pterostichus melanarius* is capable of burrowing into the soil [87] and also possesses a flexible life cycle, allowing successful adaptation to environmental changes [91,92]. It is sometimes regarded as an indicator of anthropogenically disturbed forests [56].

Nevertheless, these superdominant species, as well as other dominant species, are also generally common in older broadleaved forests [42,53]. Apparently, spontaneous succession following clear-cutting generally leads to the recovery of a dominant species complex typical of forests, as has also been reported in other regions [93,94].

In terms of life forms, particular attention should be paid to the occurrence of myxophytophagous species, which are predominantly associated with open habitats [75]. Broadleaved forests generally support a large number of myxophytophagous species, although these are usually represented by few individuals [42,70]. They inhabit small open patches within forests and may also migrate from surrounding open habitats. In our secondary forests, myxophytophages constitute only a small proportion overall, which corresponds to previous studies of secondary forests [42]. However, at some sites, these species become dominant or even superdominant. This is especially characteristic of *Harpalus rufipes* in forests with a high proportion of bare ground. Moderately warm habitats in the southern part of the biome may be favourable for oviposition by this species [95]; however, the food resources of such forests appear insufficiently attractive for it [96,97,98]. This species is known to possess strong dispersal abilities through walking migrations [99,100], and cases of high activity density in transit habitats due to migration have been reported [101]. Therefore, it cannot be excluded that secondary forests serve not only as residential habitats but also as transit habitats for this species.

### 4.2. Differentiation of Ground Beetle Assemblages

Observed differences among ground assemblages are explained only to a limited extent by present-day vegetation characteristics. The abundance of hazel (*Corylus avellana*) was found as the most robust predictor of carabid assemblages, both in terms of species composition and species structure. Hazel effectively shades the ground layer, somewhat dries the soil, and limits the growth of other plant species [44]. The presence of ash (*Fraxinus excelsior*) in the canopy and regeneration layer indicates favourable thermal conditions and fertile soils. The presence of oak (*Quercus robur*) in the canopy, and especially in the regeneration layer, indicates greater light availability [44]. However, a leave-one-site-out sensitivity analysis showed that plots with very high or low percentages of these tree species are excluded among secondary forests, and after their removing we cannot estimate these factors as significant for ground beetle assemblages among secondary forests as a whole.

The influence of surrounding landscapes on assemblages of carabids in secondary forests is confirmed by the negative correlation between the activity density of epigeobionts and the distance to large forest tracts. It indicates the importance of migrations from these source habitats in maintaining ground beetle assemblages in secondary forests. Obviously, linear distance is a very coarse measure of habitat connectivity, and a more detailed assessment of landscape matrix quality is required to evaluate the success of ground beetle migrations among habitat patches [84,102].

### 4.3. Limitations

**Pitfall trapping.** The relationship between pitfall trap catches and the actual population density and community structure of carabid beetles has long been debated by entomologists [103,104,105,106]. It is well established that pitfall trap data do not measure absolute density but rather relative abundance (activity density), which depends not only on the true abundance of beetles but also on their activity levels. Surface activity varies among species (e.g., between small and large species) and changes throughout the season. Moreover, some carabid species are poorly sampled or not captured at all by pitfall traps. Nevertheless, pitfall trapping remains the most widely used method for studying ground beetles. Therefore, our results are broadly comparable with those of most previous studies conducted in the nemoral biome.

**Number of traps.** In this study, we used ten pitfall traps per sampling plot. This number is commonly used in carabid studies [42,107]. However, it has been shown that, in low-disturbance nemoral forests, an adequate assessment of the species composition of a single habitat requires at least 30 pitfall traps [108]. Therefore, species richness in our secondary deciduous forests was probably underestimated, although sample coverage indicated that only very rare species remained undetected in individual sampling plots.

**Sampling period.** Pitfall traps were operated only from May to July, which probably resulted in an underestimation of the abundance of autumn-breeding species that may be common in broadleaved forests [109]. However, this period is appropriate for sampling the spring and early summer fauna, when carabid species diversity reaches its seasonal maximum in temperate forests [110]. Furthermore, some authors have suggested that even a one-month sampling period may be sufficient for biodiversity assessments in temperate forests [111].

**Interannual variability.** It is well known that the activity density of carabids in low-disturbance forests varies spatially and between years by a factor of approximately 1.3–1.5 [112]. The sampling year was included as a covariate in the statistical models. However, the sampling year was partly confounded with region because not all regions were surveyed in the same years (for example, several regions were sampled only in 2022). Consequently, the estimated effect of sampling year should be interpreted with caution, as it cannot completely disentangle temporal variation from regional differences.

## 5. Conclusions

The species composition of ground beetle assemblages varies greatly among secondary deciduous forests of the nemoral biome and is weakly predictable. There are many singletons. However, an absolute majority of specimens belong to the detected species.

In most cases, the core of ground beetle assemblages in secondary deciduous forests is formed by two forest generalists; superdominant species are relatively large beetles belonging to different groups of life forms—large walking epigeobiont zoophages and soil–litter stratobionts. The contribution of myxophytophages is relatively low, but in some plots, one myxophytophagous stratohorthobiont dominates.

Similarity among ground beetle assemblages, in terms of both species structure and species composition, is more strongly influenced by characteristics of the tree layer, shrub layer, and ground cover, as well as by factors not assessed in the present study, than by the spatial proximity of habitats. The strongest, although still relatively weak, predictor of both species structure and species composition is the percentage of *Corylus avellana* in the shrub layer.

The activity density of myxophytophages stratohortobionts is influenced by the proportion of bare ground. The activity density of large walking epigeobiont zoophages is higher near large forest tracts, and secondary forests adjoining large forest tracts may be important for maintaining populations of subendemic ground beetle species.

From a practical perspective, the results suggest that only a small fraction of the regional species pool is represented in each secondary forest. Therefore, surveys of ground beetles conducted in individual secondary forests do not allow reliable characterization of regional or even local ground beetle faunas.

## Figures and Tables

**Table 1:** A brief characteristic of sample plots.

Label	Lat	Lon	Year; Dates	Tree Canopy	Undergrowth	*Corylus*, %	Herb Cover, %		
							GS	Fb	BG
MO-Bb	54.579	43.213	2022 05.05/04.07	LOMAshAsp	AshOLM	10	0	95	5
MO-Br	54.537	42.897	2008 10.05/06.06	BOLMAsp	BLOM	15	70	20	10
MO-Ch	54.772	43.672	2019 03.05/07.07	OLBAsp	LO	5	50	50	0
MO-S1	54.194	45.133	2015 26.04/19.07	OLMAshBEAsp	OLMAshMB	10	80	15	5
MO-S2	54.197	45.135	2014 18.05/20.07	OLMAshBEAsp	LOMAshMB	10	75	25	0
MO-S3	54.193	45.129	2013 05.05/27.06	OLMAshBEAsp	OLMAshMB	10	80	15	5
MO-T	54.554	43.416	2022 05.05/04.07	OBAsp	P	0	40	60	0
PNZ-I	53.670	44.755	2022 24.05/23.07	OLMAshBE	OLMAshMB	10	50	40	10
PNZ-N	53.265	42.943	2023 06.05/07.07	O	O	0	5	10	70
PNZ-S	53.885	44.700	2022 24.05/23.07	OLMAshEB	OLMAshMB	10	50	35	15
RYA-A	53.404	39.296	2023 26.05/21.07	OLMAshBE	OLMAshMB	5	65	35	0
RYA-Ch	53.460	39.509	2022 02.05/29.07	ML	ML	0	5	75	10
RYA-K	53.523	40.155	2022 02.05/29.07	BAsp	M	0	10	85	5
RYA-M	53.465	39.245	2022 02.05/29.07	LB	LM	0	1	25	60
RYA-Ng	53.691	39.954	2023 26.05/21.07	OML	MOL	20	30	70	0
RYA-Nk	53.751	40.869	2023 26.05/21.07	OLMAshBEAsp	OLMAshMB	10	60	35	5
RYA-P	54.831	39.350	2022 20.05/21.07	LO	L	0	1	15	80
SAR-O	51.970	43.544	2022 06.05/09.07	OLMAshBE	MLOAsh	0	5	30	65
ULY-T	53.951	46.392	2022 17.05/16.07	OLMAshBE	MLAshOB	10	90	10	0
ULY-Y	53.969	46.393	2022 17.05/16.07	OLMAshBE	LOMAshMB	10	60	40	0
VOR-P	51.151	41.576	2022 03.06/08.07	AshAspL	AshLM	0	1	10	70

Notes: Ash—ash, Asp—aspen, B—birch, E—elm, L—lime, M—maple, O—oak, P—pine, GS—grasses and sedges, Fb—forbs, BG—bare ground. Tree species are listed in descending order of abundance; species with equal abundance are arranged alphabetically. See the Supplementary File for details.

**Table 2:** Occurrence (number of sampling plots) and activity density of Carabidae in secondary deciduous forests of the nemoral biome in Central Russia.

Species	Number Plots	Activity Density, Specimens per 100 Trap Days		
		Mean	Median	Max
*Acupalpus meridianus* (Linnaeus, 1761)	2	0.01	0	0.17
*Agonum duftschmidi* J.Schmidt, 1994	4	0.14	0	2.17
*Agonum gracilipes* (Duftschmid, 1812)	4	0.15	0	2.50
*Amara aenea* (De Geer, 1774)	3	0.09	0	0.74
*Amara bifrons* (Gyllenhal, 1810)	3	0.03	0	0.29
*Amara communis* (Panzer, 1797)	5	0.26	0	2.50
*Amara consularis* (Duftschmid, 1812)	1	0.01	0	0.29
*Amara convexior* Stephens, 1828	1	0.05	0	1.07
*Amara eurynota* (Panzer, 1796)	7	0.09	0	0.80
*Amara famelica* C.C.A. Zimmermann, 1832	1	0.07	0	1.48
*Amara lunicollis* Schiødte, 1837	1	0.02	0	0.37
*Amara nitida* Sturm, 1825	2	0.19	0	3.71
*Amara ovata* (Fabricius, 1792)	3	0.35	0	6.86
*Amara similata* (Gyllenhal, 1810)	4	0.05	0	0.33
*Anchomenus dorsalis* (Pontoppidan, 1763)	2	0.05	0	0.83
*Asaphidion flavipes* (Linnaeus, 1761)	2	0.02	0	0.32
*Badister bullatus* (Schrank, 1798)	6	0.19	0	2.00
*Badister lacertosus* Sturm, 1815	10	0.24	0	1.33
*Badister sodalis* (Duftschmid, 1812)	3	0.08	0	0.83
*Badister unipustulatus* Bonelli, 1813	1	0.01	0	0.17
*Bembidion guttula* (Fabricius, 1792)	1	0.04	0	0.83
*Bembidion lampros* (Herbst, 1784)	2	0.11	0	1.27
*Bembidion mannerheimii* C.R. Sahlberg, 1827	3	0.15	0	2.06
*Bembidion quadrimaculatum* (Linnaeus, 1761)	1	0.01	0	0.16
*Brachinus elegans* Chaudoir, 1842	1	0.01	0	0.11
*Calathus fuscipes* (Goeze, 1777)	1	0.20	0	4.29
*Calathus melanocephalus* (Linnaeus, 1758)	1	0.01	0	0.29
*Calathus micropterus* (Duftschmid, 1812)	1	0.01	0	0.17
*Calosoma inquisitor* (Linnaeus, 1758)	10	0.63	0	7.00
*Calosoma investigator* (Illiger, 1798)	1	0.01	0	0.17
*Carabus cancellatus* Illiger, 1798	19	11.11	4.83	42.00
*Carabus convexus* Fabricius, 1775	3	0.33	0	6.57
*Carabus estreicheri* Fischer von Waldheim, 1820	1	0.01	0	0.12
*Carabus granulatus* Linnaeus, 1758	12	4.73	0.34	35.33
*Carabus hortensis* Linnaeus, 1758	3	0.04	0	0.50
*Carabus marginalis* Fabricius, 1794	2	0.62	0	12.57
*Carabus nemoralis* O.F. Müller, 1764	5	0.88	0	10.16
*Carabus schoenherri* Fischer von Waldheim, 1820	2	0.02	0	0.17
*Carabus stscheglowi* Mannerheim, 1827	2	0.27	0	5.14
*Carabus violaceus aurolimbatus* Dejean, 1830	2	0.01	0	0.11
*Chlaenius nigricornis* (Fabricius, 1787)	1	0.01	0	0.11
*Clivina fossor* (Linnaeus, 1758)	2	0.01	0	0.16
*Cymindis angularis* Gyllenhal, 1810	1	0.01	0	0.17
*Harpalus distinguendus* (Duftschmid, 1812)	4	0.90	0	18.00
*Harpalus griseus* (Panzer, 1796)	6	0.57	0	7.14
*Harpalus hirtipes* (Panzer, 1796)	1	0.01	0	0.11
*Harpalus laevipes* Zetterstedt, 1828	2	0.02	0	0.33
*Harpalus latus* (Linnaeus, 1758)	7	0.13	0	0.83
*Harpalus progrediens* Schauberger, 1922	2	0.02	0	0.17
*Harpalus pumilus* Sturm, 1818	2	0.05	0	0.74
*Harpalus rubripes* (Duftschmid, 1812)	5	0.06	0	0.38
*Harpalus rufipes* (De Geer, 1774)	14	8.41	0.47	70.83
*Harpalus signaticornis* (Duftschmid, 1812)	5	0.04	0	0.17
*Harpalus smaragdinus* (Duftschmid, 1812)	1	0.03	0	0.57
*Harpalus tardus* (Panzer, 1796)	4	1.15	0	21.71
*Harpalus xanthopus winkleri* Schauberger, 1923	5	0.07	0	0.50
*Leistus ferrugineus* (Linnaeus, 1758)	2	0.01	0	0.17
*Licinus depressus* (Paykull, 1790)	2	0.10	0	2.00
*Limodromus krynickii* (Sperk, 1835)	3	0.19	0	3.50
*Limodromus assimilis* (Paykull, 1790)	10	6.98	0	43.50
*Loricera pilicornis* (Fabricius, 1775)	3	0.06	0	0.67
*Nothiophilus aestuans* Dejean, 1826	1	0.01	0	0.17
*Notiophilus aquaticus* (Linnaeus, 1758)	1	0.02	0	0.50
*Notiophilus germinyi* Fauvel, 1863	3	0.04	0	0.36
*Notiophilus palustris* (Duftschmid, 1812)	9	0.25	0	1.33
*Ophonus azureus* (Fabricius, 1775)	1	0.01	0	0.17
*Ophonus laticollis* Mannerheim, 1825	1	0.01	0	0.11
*Ophonus rufibarbis* (Fabricius, 1792)	4	0.10	0	1.70
*Ophonus schaubergerianus* (Puel, 1937)	1	0.01	0	0.14
*Oxypselaphus obscurus* (Herbst, 1784)	4	0.08	0	0.75
*Panagaeus bipustulatus* (Fabricius, 1775)	10	0.25	0	1.43
*Patrobus assimilis* Chaudoir, 1844	1	0.01	0	0.17
*Poecilus cupreus* (Linnaeus, 1758)	5	0.15	0	1.32
*Poecilus lepidus* (Leske, 1785)	2	0.05	0	0.74
*Poecilus versicolor* (Sturm, 1824)	14	0.77	0.17	8.00
*Pterostichus anthracinus* (Illiger, 1798)	2	0.02	0	0.33
*Pterostichus melanarius* (Illiger, 1798)	19	6.44	4.62	24.20
*Pterostichus minor* (Gyllenhal, 1827)	3	0.04	0	0.33
*Pterostichus niger* (Schaller, 1783)	12	0.65	0.15	4.00
*Pterostichus nigrita* (Paykull, 1790)	4	0.06	0	0.50
*Pterostichus oblongopunctatus* (Fabricius, 1787)	17	2.06	0.91	18.67
*Pterostichus quadrifoveolatus* Letzner, 1852	1	0.01	0	0.17
*Pterostichus strenuus* (Panzer, 1796)	14	0.55	0.16	3.49
*Pterostichus uralensis* (Motschulsky, 1850)	1	0.06	0	1.17
*Pterostichus vernalis* (Panzer, 1796)	1	0.01	0	0.17
*Stenolophus mixtus* (Herbst, 1784)	1	0.01	0	0.17
*Stomis pumicatus* (Panzer, 1796)	6	0.16	0	1.33
*Synuchus vivalis* (Illiger, 1798)	1	0.01	0	0.17
*Trechus secalis* (Paykull, 1790)	2	0.04	0	0.67
*Zabrus tenebrioides* (Goeze, 1777)	1	0.01	0	0.29

**Table 3:** Structural characteristics of ground beetle assemblages in secondary deciduous forests of the nemoral biome.

Plot	Number of Species		Activity Density	Shannon H	Berger–Parker
	All	≥4 Specimens			
MO-Bb	19	6	93.7	1.66	0.464
MO-Br	9	3	10.4	2.17	0.214
MO-Ch	14	3	6.2	2.444	0.250
MO-S1	20	5	14.2	2.247	0.395
MO-S2	21	11	114.3	1.955	0.301
MO-S3	15	7	33.6	2.006	0.371
MO-T	19	4	41.0	1.307	0.655
PNZ-I	13	4	19.0	1.772	0.368
PNZ-N	13	3	16.8	1.787	0.327
PNZ-S	32	8	109.0	1.38	0.650
RYA-A	11	1	11.2	2.093	0.414
RYA-Ch	14	7	19.5	1.91	0.430
RYA-K	18	6	63.6	1.337	0.621
RYA-M	23	10	77.5	1.665	0.430
RYA-Ng	13	6	70.0	1.912	0.281
RYA-Nk	14	4	10.9	2.089	0.361
RYA-P	20	9	81.0	1.463	0.519
SAR-O	10	3	6.4	2.098	0.342
ULY-T	19	9	127.3	1.764	0.292
ULY-Y	19	8	29.5	2.231	0.294
VOR-P	26	16	137.1	2.434	0.265
Median	18	6	33.6	1.912	0.368

**Table 4:** PERMANOVA results for the dissimilarity of ground beetle assemblages based on the binary Jaccard index. Significant *p*-values are shown in bold.

Variable	Df	R^2^	F	*p*	Leave-One-Site-Out R^2^	Leave-One-Site-Out *p*
Model 1—with Year						
Oak in the tree layer	1	0.089	2.251	**0.001**	0.057–0.100	0.001–0.075
Ash in the tree layer	1	0.070	1.766	**0.008**	0.032–0.077	0.001–0.867
Ash in the undergrowth	1	0.053	1.348	0.087	0.048–0.061	0.029–0.241
Oak in the undergrowth	1	0.067	1.695	**0.015**	0.0470–0.077	0.004–0.389
*Corylus*	1	0.094	2.368	**0.001**	0.077–0.103	**0.001–0.005**
Year	5	0.269	1.357	**0.007**	0.221–0.292	0.003–0.051
Residuals	9	0.357				
Total	19	1				
Model 2—without Year						
Oak in the tree layer	1	0.082	1.8403	**0.002**	0.0595–0.0963	0.001–0.152
Ash in the tree layer	1	0.072	1.6177	**0.010**	0.0461–0.0786	0.005–0.539
Ash in the undergrowth	1	0.064	1.4402	**0.034**	0.0596–0.0726	0.017–0.165
Oak in the undergrowth	1	0.068	1.5191	**0.029**	0.0494–0.0800	0.010–0.401
*Corylus*	1	0.105	2.3389	**0.001**	0.0925–0.113	**0.001–0.003**
Residuals	14	0.626				
Total	19	1				
Model 3—with climatic conditions						
Oak in the tree layer	1	0.054	1.193	0.205		
Ash in the tree layer	1	0.073	1.608	**0.008**		
Oak in the undergrowth	1	0.059	1.300	0.123		
*Corylus*	1	0.105	2.313	**0.001**		
May and June temperatures	1	0.044	0.977	0.500		
May and June precipitation	1	0.037	0.820	0.740		
Residuals	13	0.590				
Total	19	1				

Leave-one-site-out results are presented as the range of partial R^2^ and *p*-values obtained after sequential exclusion of each sampling site.

**Table 5:** PERMANOVA results for the dissimilarity of ground beetle assemblages calculated using the abundance-based Jaccard index. Significant *p*-values are shown in bold.

Variable	Df	R^2^	F	*p*	Leave-One-Site-Out R^2^	Leave-One-Site-Out *p*
Model 1—with Year						
*Corylus*	1	0.084	1.690	**0.019**	0.0764–0.104	**0.001–0.026**
Oak in the undergrowth	1	0.072	1.452	0.082	0.0602–0.0892	0.007–0.224
Year	5	0.238	0.959	0.631	0.217–0.287	0.068–0.363
Residuals	12	0.594				
Total	19	1				
Model 2—without Year						
Corylus	1	0.099	2.033	**0.003**	0.0917–0.117	**0.001–0.007**
Oak in the undergrowth	1	0.102	2.091	**0.007**	0.0567–0.0821	0.022–0.340
Residuals	17	0.832				
Total	19	1				

Note: see Table 3.

## Data Availability

The original contributions presented in this study are included in the article. Further inquiries can be directed to the corresponding author.

## Supplementary Materials

The following supporting information can be downloaded at https://www.mdpi.com/article/10.3390/insects17070724/s1: Table S1: Description of sample plots; Table S2: Data on the activity density of carabids across sample plots. Table S3: The results of a leave-one-site-out sensitivity analysis.
