# Supplementary material for: Assemblages of Ground Beetles (Coleoptera, Carabidae) in Secondary Deciduous Forests of Selected Regions of the Nemoral Biome in European Russia (Spring—Early Summer Aspect)
Source: Insects. 2026 Jul 13;17(7):724. doi: 10.3390/insects17070724 (PMC13409739; doi:10.3390/insects17070724)
Supplement: Supplementary file 1 [file insects-17-00724-s001.zip › TableS3 The results of a leave.pdf]

Supplementary Table S3. The results of a leave-one-site-out sensitivity analysis of ground beetle assemblages in secondary deciduous forests of the Nemoral biome

1. Matrix based on binary Jaccard index

1.1. Model "Year + oak_t + ash_tree + ash_un + oak_un"

	Removed_site	Term	Df	F	R2	Pr(>F)
1	MO-S1	Year	4	1.308	0.221	0.022
2	MO-S2	Year	4	1.359	0.229	0.015
3	MO-S3	Year	4	1.410	0.234	0.010
4	MO-Ch	Year	4	1.419	0.236	0.003
5	RYA-Ng	Year	5	1.416	0.292	0.003
6	RYA-M	Year	5	1.391	0.288	0.008
7	ULY-Y	Year	5	1.388	0.284	0.006
8	MO-T	Year	5	1.307	0.282	0.020
9	PNZ-I	Year	5	1.413	0.282	0.003
10	RYA-A	Year	5	1.345	0.282	0.012
11	RYA-K	Year	5	1.338	0.281	0.018
12	RYA-Ch	Year	5	1.289	0.281	0.019
13	SAR-O	Year	5	1.313	0.281	0.013
14	RYA-Nk	Year	5	1.379	0.279	0.005
15	PNZ-S	Year	5	1.394	0.279	0.007
16	MO-Bb	Year	5	1.358	0.278	0.006
17	RYA-P	Year	5	1.297	0.276	0.011
18	ULY-T	Year	5	1.300	0.276	0.018
19	VOR-P	Year	5	1.224	0.273	0.051
20	PNZ-N	Year	5	1.270	0.270	0.039

	Removed_site	Term	Df	F	R2	Pr(>F)
1	MO-T	oak_t	1	1.355	0.059	0.075
2	RYA-A	oak_t	1	1.739	0.073	0.006
3	PNZ-N	oak_t	1	1.763	0.075	0.003
4	RYA-Nk	oak_t	1	2.472	0.100	0.001
5	MO-Bb	oak_t	1	2.439	0.100	0.001
6	RYA-M	oak_t	1	2.373	0.098	0.001
7	SAR-O	oak_t	1	2.281	0.097	0.001
8	VOR-P	oak_t	1	2.157	0.096	0.001
9	RYA-Ch	oak_t	1	1.947	0.085	0.001
10	PNZ-S	oak_t	1	2.389	0.096	0.001
11	MO-S1	oak_t	1	2.251	0.095	0.001
12	MO-S2	oak_t	1	2.251	0.095	0.001
13	ULY-Y	oak_t	1	2.300	0.094	0.001
14	RYA-Ng	oak_t	1	2.275	0.094	0.001
15	MO-Ch	oak_t	1	2.251	0.094	0.002
16	MO-S3	oak_t	1	2.251	0.094	0.001
17	PNZ-I	oak_t	1	2.331	0.093	0.001
18	ULY-T	oak_t	1	2.184	0.093	0.001
19	RYA-K	oak_t	1	2.110	0.089	0.001
20	RYA-P	oak_t	1	2.097	0.089	0.003

	Removed_site	Term	Df	F	R2	Pr(>F)

1	VOR-P	ash_tree	1	0.725	0.032	0.867
2	MO-Bb	ash_tree	1	1.187	0.049	0.217
3	RYA-Nk	ash_tree	1	1.908	0.077	0.001
4	RYA-Ng	ash_tree	1	1.858	0.077	0.001
5	RYA-M	ash_tree	1	1.847	0.076	0.004
6	ULY-Y	ash_tree	1	1.853	0.076	0.002
7	RYA-A	ash_tree	1	1.790	0.075	0.001
8	MO-S1	ash_tree	1	1.766	0.075	0.008
9	MO-S2	ash_tree	1	1.766	0.074	0.002
10	SAR-O	ash_tree	1	1.734	0.074	0.001
11	PNZ-N	ash_tree	1	1.728	0.074	0.005
12	MO-Ch	ash_tree	1	1.766	0.074	0.006
13	MO-S3	ash_tree	1	1.766	0.073	0.006
14	RYA-K	ash_tree	1	1.744	0.073	0.009
15	ULY-T	ash_tree	1	1.711	0.073	0.009
16	PNZ-I	ash_tree	1	1.801	0.072	0.001
17	RYA-P	ash_tree	1	1.673	0.071	0.006
18	MO-T	ash_tree	1	1.611	0.070	0.022
19	PNZ-S	ash_tree	1	1.768	0.071	0.006
20	RYA-Ch	ash_tree	1	1.611	0.070	0.013

	Removed_site	Term	Df	F	R2	Pr(>F)
1	MO-Bb	ash_un	1	1.173	0.048	0.241
2	MO-T	ash_un	1	1.183	0.051	0.220
3	RYA-Ng	ash_un	1	1.464	0.060	0.029
4	ULY-Y	ash_un	1	1.448	0.059	0.042
5	RYA-Nk	ash_un	1	1.447	0.059	0.036
6	PNZ-I	ash_un	1	1.346	0.054	0.084
7	SAR-O	ash_un	1	1.353	0.058	0.069
8	ULY-T	ash_un	1	1.282	0.054	0.135
9	MO-S1	ash_un	1	1.348	0.057	0.074
10	MO-S2	ash_un	1	1.348	0.057	0.081
11	RYA-P	ash_un	1	1.297	0.055	0.099
12	RYA-A	ash_un	1	1.349	0.056	0.086
13	RYA-Ch	ash_un	1	1.297	0.056	0.110
14	PNZ-N	ash_un	1	1.321	0.056	0.094
15	RYA-M	ash_un	1	1.359	0.056	0.081
16	VOR-P	ash_un	1	1.245	0.056	0.159
17	MO-Ch	ash_un	1	1.348	0.056	0.077
18	RYA-K	ash_un	1	1.336	0.056	0.094
19	PNZ-S	ash_un	1	1.394	0.056	0.077
20	MO-S3	ash_un	1	1.348	0.056	0.090

	Removed_site	Term	Df	F	R2	Pr(>F)
1	VOR-P	oak_un	1	1.051	0.047	0.389
2	MO-T	oak_un	1	1.237	0.053	0.195
3	ULY-Y	oak_un	1	1.871	0.077	0.004
4	SAR-O	oak_un	1	1.443	0.062	0.046
5	MO-S1	oak_un	1	1.694	0.072	0.015
6	MO-S2	oak_un	1	1.694	0.071	0.007

7	PNZ-N	oak_un	1	1.673	0.071	0.010
8	RYA-M	oak_un	1	1.714	0.071	0.011
9	RYA-K	oak_un	1	1.688	0.071	0.017
10	MO-Bb	oak_un	1	1.566	0.064	0.021
11	MO-Ch	oak_un	1	1.694	0.071	0.012
12	MO-S3	oak_un	1	1.694	0.070	0.016
13	RYA-P	oak_un	1	1.647	0.070	0.020
14	RYA-Ch	oak_un	1	1.607	0.070	0.017
15	RYA-Ng	oak_un	1	1.693	0.070	0.012
16	RYA-A	oak_un	1	1.552	0.065	0.037
17	ULY-T	oak_un	1	1.641	0.070	0.034
18	RYA-Nk	oak_un	1	1.699	0.069	0.018
19	PNZ-S	oak_un	1	1.696	0.068	0.018
20	PNZ-I	oak_un	1	1.693	0.068	0.017

2. Matrix base on abundance-based Jaccard index

2.1. Model “Year + Corylus + oak_un”

	Removed_site	Term	Df	F	R2	Pr(>F)
1	MO-S1	Year	4	1.087	0.217	0.233
2	MO-Ch	Year	4	1.133	0.224	0.138
3	MO-S2	Year	4	1.133	0.226	0.146
4	MO-S3	Year	4	1.150	0.226	0.111
5	PNZ-N	Year	5	1.165	0.287	0.084
6	RYA-Ng	Year	5	1.128	0.285	0.116
7	VOR-P	Year	5	1.175	0.284	0.068
8	MO-T	Year	5	1.179	0.284	0.076
9	PNZ-I	Year	5	1.167	0.282	0.092
10	RYA-M	Year	5	1.102	0.278	0.195
11	MO-Bb	Year	5	1.130	0.278	0.139
12	SAR-O	Year	5	1.088	0.278	0.213
13	RYA-A	Year	5	1.148	0.278	0.124
14	RYA-K	Year	5	1.104	0.274	0.201
15	RYA-Ch	Year	5	1.094	0.274	0.217
16	ULY-Y	Year	5	1.096	0.274	0.205
17	RYA-Nk	Year	5	1.028	0.260	0.363
18	RYA-P	Year	5	1.088	0.271	0.204
19	ULY-T	Year	5	1.076	0.270	0.242
20	PNZ-S	Year	5	1.097	0.269	0.198

	Removed_site	Term	Df	F	R2	Pr(>F)
1	SAR-O	oak_un	1	1.177	0.060	0.224
2	RYA-A	oak_un	1	1.843	0.089	0.007
3	VOR-P	oak_un	1	1.778	0.086	0.011
4	PNZ-N	oak_un	1	1.485	0.073	0.045
5	MO-T	oak_un	1	1.742	0.084	0.015
6	RYA-P	oak_un	1	1.472	0.073	0.069
7	ULY-Y	oak_un	1	1.670	0.083	0.016
8	RYA-Ch	oak_un	1	1.485	0.074	0.044
9	MO-S1	oak_un	1	1.623	0.081	0.033
10	MO-Bb	oak_un	1	1.645	0.081	0.017

11	MO-S2	oak_un	1	1.623	0.081	0.026
12	PNZ-I	oak_un	1	1.578	0.076	0.034
13	RYA-M	oak_un	1	1.515	0.077	0.043
14	MO-Ch	oak_un	1	1.623	0.080	0.023
15	RYA-Nk	oak_un	1	1.522	0.077	0.036
16	MO-S3	oak_un	1	1.623	0.080	0.025
17	ULY-T	oak_un	1	1.551	0.078	0.042
18	RYA-K	oak_un	1	1.577	0.078	0.046
19	RYA-Ng	oak_un	1	1.559	0.079	0.020
20	PNZ-S	oak_un	1	1.595	0.078	0.022

	Removed_site	Term	Df	F	R2	Pr(>F)
1	RYA-Ng	Corylus	1	1.515	0.076	0.026
2	SAR-O	Corylus	1	1.564	0.080	0.019
3	PNZ-I	Corylus	1	2.160	0.104	0.001
4	ULY-T	Corylus	1	1.779	0.089	0.005
5	PNZ-S	Corylus	1	2.114	0.104	0.001
6	MO-T	Corylus	1	2.108	0.101	0.001
7	MO-S1	Corylus	1	2.024	0.101	0.001
8	MO-S2	Corylus	1	2.024	0.101	0.001
9	VOR-P	Corylus	1	2.086	0.101	0.001
10	RYA-P	Corylus	1	2.008	0.100	0.001
11	MO-Ch	Corylus	1	2.024	0.100	0.001
12	RYA-K	Corylus	1	2.012	0.100	0.001
13	ULY-Y	Corylus	1	1.873	0.094	0.003
14	PNZ-N	Corylus	1	1.904	0.094	0.001
15	MO-S3	Corylus	1	2.024	0.100	0.001
16	RYA-A	Corylus	1	1.939	0.094	0.001
17	RYA-M	Corylus	1	1.968	0.099	0.001
18	RYA-Ch	Corylus	1	1.978	0.099	0.002
19	RYA-Nk	Corylus	1	1.943	0.098	0.003
20	MO-Bb	Corylus	1	1.989	0.098	0.001

2.2. Model "Corylus + oak_un"

	Removed_site	Term	Df	F	R2	Pr(>F)
1	SAR-O	oak_un	1	1.079	0.057	0.340
2	RYA-A	oak_un	1	1.621	0.082	0.030
3	VOR-P	oak_un	1	1.605	0.082	0.022
4	PNZ-N	oak_un	1	1.291	0.067	0.145
5	RYA-P	oak_un	1	1.331	0.068	0.085
6	RYA-M	oak_un	1	1.307	0.068	0.117
7	MO-S3	oak_un	1	1.509	0.077	0.033
8	RYA-Ch	oak_un	1	1.348	0.069	0.097
9	MO-Ch	oak_un	1	1.489	0.076	0.050
10	ULY-Y	oak_un	1	1.467	0.075	0.051
11	MO-S1	oak_un	1	1.473	0.075	0.062
12	MO-S2	oak_un	1	1.374	0.071	0.078
13	RYA-Nk	oak_un	1	1.462	0.075	0.044
14	RYA-Ng	oak_un	1	1.420	0.075	0.060
15	PNZ-S	oak_un	1	1.469	0.074	0.052

16	MO-Bb	oak_un	1	1.446	0.074	0.054
17	MO-T	oak_un	1	1.456	0.074	0.064
18	ULY-T	oak_un	1	1.436	0.074	0.060
19	PNZ-I	oak_un	1	1.421	0.072	0.064
20	RYA-K	oak_un	1	1.413	0.072	0.071
